# Continuing Professional Development – Radiation Therapy

**DOI:** 10.1002/jmrs.70056

**Published:** 2026-01-10

Maximise your continuing professional development (CPD) by reading the selected article and answering the five questions. Please remember to self‐claim your CPD and retain your supporting evidence. Answers will be available via the QR code and published in JMRS – Volume 73, Issue 4, December 2026.

## Magnetic Resonance Guided Radiation Therapy (MRgRT) Prostate Motion and Margins

Sammi Peng, Tegan Courtot, Jessica Lye, Farshad Foroudi, Mark Tacey, Daryl Lim Joon, Michael Chao, Ee Siang Choong, https://doi.org/10.1002/jmrs.70033.

1. Which factor is the main contributor to prostate motion during MR‐guided radiotherapy (MRgRT)?
  - Variations in magnetic field strength
  - Bladder filling and rectal gas movement
  - Patient anxiety
  - Respiratory motion
2. During adaptive MRgRT, why is real‐time motion monitoring particularly critical during the adaptation phase?
  - Prolonged contour adjustment and plan recalculation allow natural organ shift.
  - Rapid beam‐on delivery causes sudden prostate shifts.
  - Fusion planning during adaptation deforms prostate.
  - Respiratory motion peaks during adaptation.
3. How does cine‐MRI motion monitoring improve treatment safety and accuracy during MRgRT?
  - Reduces MR scan time.
  - Repositions CTV in real time to maintain coverage.
  - Adjusts radiation dose dynamically during beam‐on.
  - Allows treatment interruption if displacement exceeds PTV margins.
4. Which MRgRT feature best enables precise prostate dose delivery while minimising toxicity to surrounding organs?
  - Higher dose rates per fraction.
  - Faster image acquisition sequences
  - Superior soft tissue visualisation and organ delineation
  - No image registration required
5. Based on the findings of this study, what margins could be considered most appropriate for prostate MRgRT?
  - 1–2 mm isotropic margins
  - 5–8 mm isotropic margins
  - 2–3 mm non‐isotropic margins
  - 8–10 mm non‐isotropic margins

## Answers


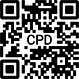
Scan this QR code to find the answers.
